# Sepsis after non‐perforated acute appendicitis

**DOI:** 10.1002/ccr3.2030

**Published:** 2019-02-06

**Authors:** Mojgan Faraji‐Goodarzi

**Affiliations:** ^1^ Faculty of Medicine, Department of Pediatrics Lorestan University of Medical Sciences Khorramabad Iran

**Keywords:** acute appendicitis, children, sepsis

## Abstract

Appendicitis is a worm‐like appendage whose base is located on the posterior side of the inner cecum. Acute appendicitis is uncommon in children younger than 5 years old. The patient was a 1.5‐year‐old boy who was admitted to the hospital with a primary complaint of fever, diarrhea, and vomiting. A pathological report of acute puffiness appendicitis with peri appendicitis was confirmed. After two weeks of treatment, the patient was discharged with an good general condition.

## 1 INTRODUCTION

Acute appendicitis in children is a serious condition and, if delayed in diagnosis, is life‐threatening. In children younger than 5 years of age, there is an unusual clinical presentation. Acute appendicitis is rare in children (1‐2 in 10 000 children younger than 4 years).

Rapid diagnosis and treatment are necessary to prevent serious complications such as abscess, perforation, obstruction, sepsis, and peritonitis. Overlapping its symptoms with other childhood conditions, including gastroenteritis, increases the likelihood of errors and delays in diagnosis.^1^

Appendicitis is a worm‐like appendage whose base is located on the posterior side of the inner cecum. Although acute appendicitis is infrequent in infants (because its base is broad and has less chance of lumen obstruction) and children younger than 5 years, however a few rare neonatal and prenatal cases have been documented.^2^, ^3^, ^4^, ^5^ The pattern of incidence of acute appendicitis indicates general tendencies to increases from early childhood until late teens before gradually declining in the adult stage. Studies have also revealed wide variation in the prevalence of appendicitis across sex, race, socioeconomic considerations.^6^, ^7^ It is more common in males. The pathophysiology of acute appendicitis is unknown, and it is a multifactorial disease. It is lower in western countries, and it raises the role of health, nutrition, and environmental factors.^8^ Overall trend analysis of the occurrence of appendicitis suggests a decline which has been attributed to improved understanding and awareness of the role of diet,^9^ hygiene,^10^ breastfeeding,^11^ genetics and environmental influences,^12^, ^13^, ^14^ and healthcare delivery.^15^ Clinical manifestations are not specific in different children. The most common symptom is abdominal pain. About 1%‐8% of children with abdominal pain have acute appendicitis.^8^ In children younger than 3 years, fever, diarrhea, and vomiting are the most common symptoms of appendicitis. In many cases, acute appendicitis is often confused with gastroenteritis because diarrhea occurs in 33%‐46% of cases. Other differential diagnosis such as enucleation, lower lobe pneumonia, acute pyelonephritis should be considered.^16^ Delay in diagnosis increases the risk of complications and causes morbidity and mortality in children and prolongs the duration of hospitalization. Complications include abscess, peritonitis, sepsis, perforation, and obstruction.^8^, ^17^

## 2 CASE REPORT

The patient was a 1.5‐year‐old boy who was admitted to the hospital with a primary complaint of fever, diarrhea, and vomiting. At the beginning of the hospitalization, the patient had a generalized clonic‐tonic seizure. The patient was dispatched to our intensive care unit on the fourth day of admission due to reduced consciousness.

Patient is the first child of a family and is resident of the village. He had a history of pneumonia at 6 months of age. There was a history of seizure of fever in his family. From the outset, he was treated with ceftriaxone, vancomycin, phenytoin, and acyclovir. The growth and development were normal.

The patient had tachypnea and tachycardia, and fever was 39°C. Percent oxygen saturation without getting oxygen was 98%. It was toxic and had GCS = 8. The patient had a mild tenderness on the right side of the abdomen with a predominance in the RLQ (right lower quadrant). The rectal examination was normal. The amount of urine output was normal. Patients were treated with liquid therapy, and intravenous antibiotics were changed to moropenem and vancomycin. Patient tests: serum electrolytes (sodium and potassium), blood gas analysis, coagulation tests, liver and kidney function tests, vidal test and Albumin were normal.

Some of the patient's laboratory results were as follows:

$$\begin{array}{c} \text{S}/\text{C}=\text{NO growth,}\;\;\text{S}/\text{E:WBC =}\;20-30,\;\;\text{RBC = MANY / ESR =}\;74,\;\text{CRP = +}\;,\;\text{WBC =}\;1100,\\ \;\text{POLY =}\;49.9\%,\;\text{LYMPH =}\;40.1\%,\;\text{Band =}\;13\%,\;\text{HB =}\;10.2,\;\text{MCV =}\;80,\;\text{PLT =}\;45\;000 \end{array}$$

In addition, he had hypocalcemia and Hypomagnesemia and were treated with sepsis as a common symptom. Due to biliary secretion from the stomach tube, bloody diarrhea, tenderness and abdominal distension, abdominal ultrasonography was performed with suspicion of obstructive problems such as enuresis and acute appendicitis.

**Figure 1:**
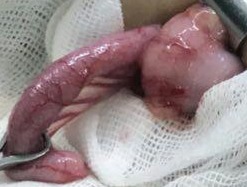
Patient Appendectomy: An inflamed appendix with a length of 8 cm and a diameter of 0.5 cm, with fibrin and exudate levels

Ultrasound findings favored the diagnosis of acute appendicitis: insignificant free fluid in the space between the interleuk and a non‐compressible appendix of 65 mm in the lower right quadrant of the abdomen was evident. The patient underwent appendectomy (Figure 1). One day after the surgery, the child was alert (Glasgow Coma Scale /Score GCS = 12), but the fever continued. Metronidazole and intraperitoneal ciprofloxacin were administered. Two days after the commencement of these antibiotics and appendectomy, the patient's alertness became normal, and the general condition was satisfactory with erythrocyte sedimentation reaction (ESR) = 32, and platelet count and white blood cell count increased. A pathological report of acute puffiness appendicitis with peri appendicitis confirmed. After two weeks, the patient was discharged with a good general condition.

## 3 DISCUSSION

Complete blood count (CBC), C‐reactive protein (CRP), urine analysis (U/A) were performed for the patient. The WBC had low sensitivity and specificity. In 20% of cases, the number of blood leukocytes in acute appendicitis is normal. CRP is more specific to the number of leukocytes. procalcitonin in the diagnosis of appendicitis is not useful. Abdominal CT scan is the most sensitive method for diagnosis of appendicitis, but because of its high radiation, physicians use abdominal ultrasound as the first choice. Abdominal CT scan is highly sensitive and specific. Intestinal gases, obesity, abdominal tenderness do not interfere with the quality of images.^16^ Diagnosis of acute appendicitis in young children is particularly complex involving several biochemical assays. But more importantly is the use of advanced imaging diagnostic tools such as abdominal X‐ray,^18^ Ultrasonography (USG),^19^ Computed Tomography Scan (CT scan) and MRI.^20^ Acute appendicitis requires urgent surgical intervention. The goal is rapid diagnosis and surgery before perforation. In rare cases, bacteremia and sepsis occur following acute non‐perforated appendicitis. In the study conducted by Nishizawa et al,^21^ an Ecoli induced sepsis in a patient with a normal immune system was detected and appendectomy revealed appendicitis without perforation. In our patient, the non‐perforated appendix was also due to seizure delay due to diagnosis. The ease of perforation in this age group is associated with some peculiar anatomical features such as thin‐walled appendix and inadequate omental barrier. A recent report has pegged the rate of missed diagnosis in children of 3 years and below at 70%‐100% and further revealed that as age decreases, there is a 5‐fold increase in the risk of developing appendicitis complication as a consequence of misdiagnosis.^8^ Clinical manifestations of sepsis before appendectomy surgery, including abnormal body temperature, tachycardia, tachypnea, WBC < 5000 or more than 15 000, and abnormal forms of tuberculosis.^22^ The patient had all sepsis symptoms, and appendectomy had shown inflamed non‐perforated appendicitis. According to Gortani et al,^23^ Sepsis Klebsiella has been reported as a non‐perforated acute appendicitis in a 11‐year‐old child with normal immune system. However, the pathogenesis of sepsis in a non‐perforated appendix is still unknown.^24^ But it has been suggested it may occur due to the release of microorganisms from the lumen of the intestine into systemic blood flow.^23^ The greatest delay in the diagnosis of acute appendicitis occurs in children who are referred to the clinic with symptoms of acute gastroenteritis. Common causes of delayed or misdiagnosis in under 5 children is chiefly due to non‐specific clinical manifestation, inability to communicate nature and precise location of pain, inadequate examination consequently leading to perforation and other complications. A recent report has pegged the rate of missed diagnosis in children of 3 years and below at 70%‐100%, and further revealed that as age decreases, there is a 5‐fold increase in the risk of developing appendicitis complication as a consequence of misdiagnosis.^8^ Management of children diagnosed of acute appendicitis involves a number of protocols which is mainly subject to the level of complications involved at the time certainty was established. Typically, children suspected of having acute appendicitis are immediately placed on admission for a close or active monitoring and clinical examinations in addition to scoring systems depending on the myriad of the symptoms presented so as to minimize misdiagnosis.^25^, ^26^ Open appendectomy is the most common approach for children but advanced minimally invasive technique such as laparoscopy is now being introduced by skilled surgeons.^27^ Subsequent recuperation devoid of events with appropriate fluid resuscitation and antibiotic administration, a smooth and full recovery is the eventual outcome.

## 4 CONCLUSION

Acute appendicitis in children can be deadly when it diagnosis is mistaken or delayed as such rapid diagnosis and with surgical intervention can greatly reduce associated mortality and morbidity. It therefore essential that adequate attention is given to the diagnosis phase and process utilizing available imaging tools, relevant scoring approaches alongside the traditional laboratory assays to reduces incidences of missed initial diagnosis. The atypical presentation in under 5 age children is a challenge that must be handled with caution to achieve timely diagnosis and intervention. In a child with persistent fever, diarrhea and vomiting, or in cases of sepsis, with abdominal tenderness, the possibility of acute appendicitis should be considered.

## CONFLICT OF INTEREST

The authors deny any conflict of interest in any terms or by any means during the study. All the fees provided by research center fund and deployed accordingly.

## AUTHOR CONTRIBUTIONS

MF‐G: conceptualized and designed the study, drafted the initial manuscript, and reviewed and revised the manuscript; designed the data collection instruments, collected data, carried out the initial analyses, and reviewed and revised the manuscript; coordinated and supervised data collection, and critically reviewed the manuscript for important intellectual content. All authors approved the final manuscript as submitted and agree to be accountable for all aspects of the work.
